# Extraction and Microencapsulation of Bioactive Compounds from Muicle (*Justicia spicigera*) and Their Use in the Formulation of Functional Foods

**DOI:** 10.3390/foods10081747

**Published:** 2021-07-29

**Authors:** Norma Cristina Castro-Alatorre, Tzayhrí Gallardo-Velázquez, Luis Carlos Boyano-Orozco, Darío Iker Téllez-Medina, Ofelia Gabriela Meza-Márquez, Guillermo Osorio-Revilla

**Affiliations:** 1 Departamento de Ingeniería Bioquímica, Escuela Nacional de Ciencias Biológicas, Instituto Politécnico Nacional, Av. Wilfrido Massieu S/N, Col. Unidad Profesional Adolfo López Mateos, Zacatenco, CP. 07738 Mexico City, Mexico; norma_cristina94@hotmail.com (N.C.C.-A.); luisboyano8@gmail.com (L.C.B.-O.); darioiker@gmail.com (D.I.T.-M.); ogmmz@yahoo.com.mx (O.G.M.-M.); 2 Departamento de Biofísica, Escuela Nacional de Ciencias Biológicas, Instituto Politécnico Nacional, Prolongación de Carpio y Plan de Ayala S/N. Col. Santo Tomás, CP. 11340 Mexico City, Mexico

**Keywords:** *Justicia spicigera*, spray drying, antioxidant capacity, bioactive compounds, microencapsulation, functional foods

## Abstract

Bioactive compounds (BC) present in muicle leaves were extracted using the best extraction conditions obtained with a Box–Behnken experimental design, extracting 95% of BC. Microencapsulation of muicle BC was carried out by spray drying using DE10 maltodextrin (MD) and soy protein isolate (SPI) as encapsulating agents. The best conditions for the ethanolic extraction of BC from muicle were 30 °C, 40% aqueous ethanol, and one extraction for 1 h. The best spray drying encapsulating conditions for BC and antioxidant capacity (AC) using MD as an encapsulating agent were: 160–80 °C and 10% MD in the feeding solution, and for SPI: 180–70 °C and 5% SPI in the feeding solution. Microcapsules were added to yogurt and a sensory evaluation and retention of BC during 15-day storage at 4 °C was performed. Sensory evaluation showed that yogurt with added MD microcapsules had better acceptance than that with SPI microcapsules. Based on this, a jelly with added muicle MD microcapsules was also prepared which obtained better acceptance by the judges. At the end of the storage period, yogurt with SPI microcapsules showed better retention of BC and AC than yogurts with MD microcapsules; however, products with MD microcapsules had better acceptance.

## 1. Introduction

In modern times, people are more concerned about leading a healthy life, seeking the consumption of bioactive compounds or functional foods which directly impact health, such as the antioxidants found in fruits and vegetables [1].

The development of functional food products has proven to be a good marketing strategy for the industry, improving its products by the incorporation of bioactive compounds, such as antioxidants, vitamins, probiotic microorganisms, and fiber. These kinds of products are consumed by most of the population, from children to elderly people [2]. Yogurt is another product that has had bioactive compounds added to improve its functional characteristics, apart from having lactic acid bacteria that improve digestion and intestinal health [3].

Various plants contain a wide variety of bioactive compounds such as lipids, phytochemicals, aromas, fragrances, and pigments, those extracts are widely used in the food, cosmetic and pharmaceutical industries. In particular, the antioxidant activity of the extracts of certain plants is attributed mainly to the presence of phenolic compounds, volatile and non-volatile compounds, such as flavonoids, phenolic acids, and phenolic diterpenes [4,5]. 

*Justicia spicigera* (muicle or muitle as a common name) is a plant that belongs to the Acanthaceae family [6] which has been used since pre-Hispanic times in traditional Mexican medicine for the treatment of dysentery, diabetes, leukemia, and anemia. The aqueous extract of *Justicia spicigera* leaves has a dark blue color, which has been used as a food coloring as well. This extract has shown antioxidant properties due to the phenolic compounds it contains [7,8]. Muicle contains phenolic compounds which provide an antioxidant activity that inhibits the oxidation process in the body; however, these compounds when extracted, are sensitive to environmental factors such as UV radiation, oxygen, and temperature, among others. These factors affect stability and reduce the benefits of antioxidant compounds. Therefore, the protection of antioxidant compounds employing microencapsulation is important in the formulation of functional foods [9,10]. 

Microencapsulation is a technique that consists of transforming foods from liquid to solid form. It is used in the food industry to protect functional food ingredients (pigments, essential oils, oleoresins, flavors, and lipids) and bioactive compounds from evaporation, oxidation, degradation, loss of volatiles, production of foreign flavors during storage, and decrease stability and shelf life [11,12,13,14,15]. Microencapsulation by spray drying has proven to be an easy and effective technology for the protection of bioactive compounds because it has low thermal stress; and can be used with confidence to retain heat-sensitive bioactive compounds such as antioxidants, natural pigments, and vitamins [10,16]. Currently, there is only one work on microencapsulation of muicle, which focused on the stability of natural pigments during storage in mixtures of protective colloids by means of spray drying. The present work on the contrary is focused on the extraction and preservation of the bioactive compounds of muicle, not only on the natural pigment [17].

Based on the above, the aim of this work was the microencapsulation of the optimized aqueous ethanolic extract of muicle by spray drying, using DE10 maltodextrin (MD) and Isolate soy protein (SPI) as encapsulating agents, and including these microcapsules in the preparation of functional foods.

## 2. Materials and Methods 

### 2.1. Chemicals and Reagents

Potassium persulphate was obtained from Fermont (Mexico City, Mexico) Folin-Ciocalteu reagent and sodium carbonate from Meyer (México) 2-2 difenil-1-picrilhidrazilo (DPPH), 2′2-azino-bis-3-ethylbenzothiazoline-6-sulphonic acid (ABTS), potassium iodate, vanillin, 6-hydroxy,2,3,7,8-tetrametilcroman-2-carboxílico (TROLOX), tannic acid, gallic acid from Sigma-Aldrich (St. Louis, MO, USA), sodium nitrite, aluminum chloride, ethanol, and methanol from Reasol (Mexico City, Mexico). The encapsulating agents were maltodextrin (MD) with a dextrose equivalent (DE) of 10 from Amidex (Mexico City, Mexico); and the soy protein isolates 90 J (SPI) from Tres Villas (Mexico City, Mexico).

### 2.2. Plant Material

Fresh muicle (*J. spicigera*) leaves were obtained from Mercado Sonora, Mexico City. Damaged leaves were removed, and the rest were washed by immersion in water until free of soil residues, drained, and dried with absorbent paper. Leaves were dried using two different procedures: drying at room temperature (24 ± 2 °C) for 3 days away from direct sunlight, and convective fix bed drying for 4 h at 47 °C with a bed height of 20 cm. The dried material was packed in Ziploc^®^ polyethylene bags and stored at −20 °C until used.

### 2.3. Extraction of Bioactive Compounds from Muicle Leaves 

Dried muicle (*J. spicigera*) leaves were grounded using an Oster food processor (Oster, Mexico) and sieved using a 60-mesh sieve (250 µm) to homogenize particle size. Samples were extracted initially in accordance with the stirring method proposed by Baqueiro and Guerrero [7], which consists of extracting 0.3 g of powder with 25 mL water:ethanol solution (30:70% *v*/*v*) for 2 h at room temperature with stirring every 15 min. In order to quantify the total content of bioactive compounds (BC) present in muicle leaves, a series of consecutive extractions were carried out, until an absorbance less than 0.1 was obtained during the determination of phenolic compounds. This was achieved with 3 consecutive extractions. Extracts were filtered through Whatman paper No. 4 under vacuum and stored in amber glass bottles until analysis. 

### 2.4. Determination of the Best Conditions to Maximize Extraction of Bioactive Compounds from Muicle Leaves

The determination of the best condition to maximize bioactive compounds extraction from *J. spicigera* leaves was performed with a Box–Behnken experimental design (BBD); three factors were used for the experimental design: temperature (30–40 °C), ethanol concentration (0–60% *w*/*w*) and extraction time (1–2 h). The experimental matrix consisted of 26 runs with replicates in all points, to maximize the extraction of phenolic compounds (PC) and flavonoids.

### 2.5. Concentration of Bioactive Compounds Extract

In order to increase the concentration of the bioactive compounds in the extract, to carry out microencapsulation, it was necessary to concentrate the extract obtained in the optimized conditions. This was carried out in a Rotavapor^®^ R-300 (BUCHI, 106 Switzerland) at 50 °C until the final concentration was 2 mg gallic acid equivalents per gram of extract (mg GAE/g extract) similar to those reported by Robert et al. [18] for pomegranate extract and juice. Finally, the concentrated extract was filtered through Whatman paper No. 4 under vacuum.

### 2.6. Analytical Methods

#### 2.6.1. Total Phenolic Compounds 

Determination of total phenolic compounds (PC) was performed using the technique of Folin Ciocalteu described by Singlenton and Rossi [19]. In test tubes, covered with aluminum foil, 1 mL of extract was mixed with 7.5 mL of distilled water and 0.5 mL of Folin Ciocalteu 50%; after 8 min, 1.5 mL of sodium carbonate 20 %_w_ was added. The samples were kept in the dark for 60 min. A blank using 1 mL of distilled water instead of extract, was prepared. Absorbance at 750 nm was measured in a UV/VIS spectrophotometer (Jenway 632D, UK). The results were expressed in mg gallic acid equivalents per gram of extract (mg GAE/g_ext_), and in mg gallic acid equivalents per gram of dry weight (mg GAE/g_dw_) for the initial solution before drying and reconstituted powders, using a gallic acid calibration curve.

#### 2.6.2. Flavonoids Determination

Flavonoids (FL) were analyzed using the Zhishen et al. [20] method. 1 mL of sample was placed in a 10 mL volumetric flask; 4 mL distilled water, and 0.3 mL NaNO_2_ (1:20) were added and mixed for 10 s. After 6 min, 0.3 mL of AlCl_3_ (1:10) and 2 mL 1 M NaOH were added and made to the volume with distilled water. The solution was mixed, and the absorbance at 510 nm was obtained; distilled water was used as the blank. The results were expressed as milligrams of catechin equivalents per gram of extract (mg CE/g_ext_) for the optimized extract, and in milligrams of catechin equivalents per gram of dry weight (mg CE/g_dw_) for the initial solution before drying and reconstituted powders, using a catechin calibration curve. 

#### 2.6.3. Antioxidant Capacity 

Antioxidant capacity (AC) was determined using two radical inhibition assays: 

ABTS method [21]. Radical formation: 88 μL of a 140 mM potassium persulfate solution, and 5 mL of ABTS (2,2′-azino-bis-3-ethylbenzothiazoline-6-sulphonic acid) were placed in an amber bottle and left to rest in the dark for 16 h. The ABTS radical was diluted with ethyl alcohol until an absorbance of 0.700 ± 0.05 at 734 nm was obtained. Three milliliters of diluted radical ABTS was added to 0.3 mL of extract sample, mixed for 6 min, and the absorbance was measured at 734 nm. 

DPPH method [22]. A total of 2.4 mg of DPPH (2-2 difenil-1-picrilhidrazilo) were weighed and placed in a 100 mL volumetric flask, absolute methanol was added, mixed, and brought to volume; the solution was stored in amber glass. Then, 3.9 mL of radical was added to 0.1 mL of the sample and mixed for 10 s and stored in the dark at room temperature for 60 min. Absorbance was measured at 515 nm. 

Antioxidant activity by both methods was determined using a Trolox calibration curve and the inhibition percentage (%) (Equation (1). Results were expressed as micromoles of Trolox equivalents per gram of extract (µmol TE/g_ext_) for the optimized and concentrated extract, and in micromoles of Trolox equivalents per gram of dry weight (µmol TE/g_dw_) for the initial solution to be dried and reconstituted powders:

(1) $$\%\;\mathrm{Inhibition}=\frac{\mathrm{Control}\;\mathrm{Abs}-\mathrm{Sample}\;\mathrm{Abs}}{\mathrm{Control}\;\mathrm{Abs}\;}\;\times 100$$

where Abs: Absorbance.

#### 2.6.4. Condensed Tannins

Condensed tannins (CT) were determined as reported by Spranger et al. [23]. A total of 0.5 mL of the sample, 3 mL of vanillin (1%_w_), and 1.5 mL of hydrochloric acid were placed in test tubes and mixed for 10 s. After 15 min absorbance was measured at 500 nm, using a blank of ethanol. The results were expressed as milligrams of catechin equivalents per gram of extract (mg CE/g_ext_) for the optimized extract, and milligrams of catechin equivalents per gram of dry weight (mg CE/g_dw_) for the initial solution before dying and reconstituted powders, using a catechin calibration curve.

#### 2.6.5. Hydrolyzable Tannins

Hydrolyzable tannins (HT) were quantified according to Çam and Hisil [24]. Together, 1 mL of the sample and 5 mL of 2.5 %_w_ potassium iodate were placed in a test tube, mixed, and shaken for 10 s and the absorbance was measured at 550 nm; a blank of potassium iodate and water was used. The results were expressed as milligrams of tannic acid equivalents per gram of extract (mg TAE/g_ext_) for the optimized extract, and in milligrams of tannic acid equivalents per gram of dry weight (mg TAE/g_dw_) for the initial solution and reconstituted powders, using a tannic acid calibration curve. 

#### 2.6.6. Monomeric Anthocyanins

The content of monomeric anthocyanins (MA) was determined using the Wrolstad [25] method. One milliliter of the sample was placed in separate test tubes, followed by 4 mL of reagent: potassium chloride buffer (KCl) 0.025 M pH 1 in one test tube and sodium acetate buffer (NaC_2_H_3_O_2_) 0.4 M pH 4.5 in the other. They were allowed to stand for 20 min and absorbance at 510 nm and 700 nm was read. Anthocyanins content was calculated as per Wrolstad [25].

### 2.7. Preparation of Wall Material-Concentrated Extract Solution for the Spray Drying Process

#### 2.7.1. Soy Protein Isolate-Concentrated Extract Solution

An Initial soy protein isolate (SPI) solution (9%_w/v_) was prepared with distilled water, shaking the mixture until homogenized, then pH was adjusted to 9 to increase protein solubility. To remove any air bubbles present in the SPI solution, it was kept in a vacuum oven at 40 °C, for 2 h. The required SPI solution was then mixed with the concentrated muicle extract to obtain final concentrations of 5% and 7%_w/v_ of the encapsulating agent (SPI) in the solution before dying. The amount of SPI solution and muicle extract used was calculated with a mass balance.

#### 2.7.2. Maltodextrin DE10-Concentrated Extract Solution 

An initial solution of maltodextrin DE10 (MD) (30%_w/v_) was prepared with distilled water and mixed until complete solubility, then the stock solution was left to stand for 12 h at ambient temperature to eliminate air bubbles present. MD solution was then mixed with the concentrated muicle extract to obtain a final concentration of 10% and 20%_w/v_ of the encapsulating agent (MD) in the solution before drying. The amount of MD and muicle extract used was calculated with a mass balance.

### 2.8. Microencapsulation of Muicle Leaves Extract Solution

Microencapsulation of muicle extract solutions was carried out with a semi-pilot spray dryer (GEA Mobile Minor MM, Gladsaxe, Denmark) using a two-fluid external mix spray nozzle. A 2^3^ factorial design was applied; factors were: inlet air temperature, Ti (160 and 180 °C), outlet air temperature, To (70 and 80 °C), and encapsulating agent concentration (MD: 10 and 20%; SPI: 5 and 7% in the solution before drying) using as response variables: PC, F, AC, HT, and CT retention. Drying runs were performed in duplicate. Microcapsules were stored at −20 °C in sealed amber glass bottles until analysis.

### 2.9. Retention Efficiency

Retention efficiency (%RE) was evaluated by determining the BC and AC content in the solutions before drying, and in the reconstituted microcapsules per g of dry solid, expressing the result as a percentage.

(2) $$\frac{\mathrm{BC}\;\mathrm{content}\;\mathrm{in}\;\mathrm{powder}\;/g_{\mathrm{dw}}}{\mathrm{BC}\;\mathrm{content}\;\mathrm{in}\;\mathrm{solution}\;\mathrm{to}\;\mathrm{be}\;\mathrm{dried}/g_{\mathrm{dw}}}\;\times 100$$

where BC: Bioactive compounds.

### 2.10. Moisture Content Determination

Moisture content determination was carried out by placing 0.5 g of each sample in a thermobalance (Ohaus MB 200) at 110 °C until a change greater than 0.01 g was not registered during 90 s. The determination was performed in duplicate. Total solids were calculated by difference.

### 2.11. Characterization of Microcapsules Obtained under the Best Spray Drying Conditions

Particle size distribution and surface morphology were determined to the powder obtained under the best spray drying conditions for both wall materials.

#### 2.11.1. Particle Size Distribution 

Particle size and distribution were obtained with a laser diffraction particle size analyzer (IM 026 2006 series, Malvern, UK) using a 100 mm lens. Hexane (REASOL, Mexico City, Mexico) was used as a dispersant, and the particle size distribution, equivalent spherical diameter (D [4,3]), Sauter diameter (D [2,3]), and span were determined.

#### 2.11.2. Microcapsules’ Surface Morphology

Microcapsules’ surface morphology was observed from scanning electron microscope (SEM) micrographs (JSM-5800LV, Jeol, Peabody, MA, USA) obtained with an acceleration voltage of 10 kV. Microcapsules were fixed in double-faced adhesive tape stubs, coated with gold, and finally observed at 2500× magnification.

### 2.12. Preparation of Yogurt with Added Muicle Extract Microcapsules

Natural yogurt obtained from a commercial establishment was added with both: maltodextrin and soy protein isolate muicle extract microcapsules, obtained under the best drying conditions. The number of microcapsules added was enough to obtain 51 mg GAE/100 mL of product [5]. Subsequently, it was gently stirred until homogenized, finally adding the selected flavoring (coffee, because of the color it acquired with the yogurt pH).

#### Analysis of Yogurt with Added Maltodextrin or Isolate Soy Protein Microcapsules

One gram of product was weighed and brought to 5 g with distilled water at pH 10 for SPI and pH 7 for MD; 2 mL aliquots were taken, and 10 mL of ethanol were added to precipitate proteins and centrifuged at 3500 rpm for 20 min. The supernatant was taken to perform the analysis of bioactive compounds.

### 2.13. Sensory Evaluation

To determine the acceptance of the yogurt with added muicle extract microcapsules obtained with both wall materials, a 7-point hedonic test was performed [26] with three parameters: flavor aroma and appearance of the products. A total of 60 surveys were carried out for the two products with the different wall materials. These surveys were aimed at adults of both genders. After determining which wall material had better acceptance in the sensory evaluation in yogurts, a jelly was prepared as described in 2.12 with the selected microcapsules. For sensory evaluation data analysis, the total number of judges that assigned a preference on each one of the 7 points in the hedonic test for each attribute, was multiplied by the corresponding value (1–7), then the sum of all scores obtained for each attribute was divided by the total number of judges in order to obtain an average value for each attribute. For statistical analysis of the sensory evaluation results, the U Mann-Whitney test was performed.

### 2.14. Preparation of Jelly with Added Muicle Extract Microcapsules

Based on the results of the sensory evaluation of yogurt with added microcapsules, it was decided to prepare a jelly with added maltodextrin muicle extract microcapsules. Jelly preparation was adapted from Alcausa’s [27] recipe. For the preparation of jelly with maltodextrin microcapsules (50 g of product), 2.38 g of maltodextrin muicle extract microcapsules, 1.6 g of commercial gelatin, 6 g sugar, 10 drops of grape flavoring (selected because the color obtained at jelly pH 7), and 40 g of water at 40 °C were used to prepare 50 g of product. The gelatin was previously hydrated with 5 mL of water and was heated for 5 s with microwave at maximum power obtaining a solution of viscous texture. Sugar, dissolved in the remaining amount of water at (40 °C) was added; then the maltodextrin microcapsules and the flavoring were added and stirred until the complete incorporation of the ingredients. Finally, the dissolved gelatin was incorporated; all this keeping temperature at 40 °C maximum, to avoid damaging the phenolic compounds present in the product. Once all the ingredients were mixed, it was refrigerated at 4 °C until set.

#### Analysis of Jelly with Maltodextrin Muicle Extract Microcapsules

Five grams of gelatin were melted at 40 °C. A 2 mL aliquot was taken and was brought to 10 mL with absolute ethanol to precipitate the gelatin, then centrifuged at 3500 rpm for 20 min. The supernatant was taken to perform the corresponding analyzes.

### 2.15. Bioactive Compounds and Antioxidant Capacity Stability in Manufactured Functional Foods during Storage

Storage of prepared functional food products (yogurt and jelly) was carried out weekly for 15 days storage at 4 °C in absence of light, and the results expressed as % retention based on initial BC concertation at day 0.

### 2.16. Statistics Analysis

An analysis of variance (ANOVA) was applied to all data using Minitab statistical software, version 17 (Minitab Inc., State College, PA, USA).

## 3. Results and Discussion

### 3.1. Moisture Content and Remnant Phenolic Compounds in Dried Muicle Leaves

The moisture content in fresh leaves was 71.6%; leaves dried at room temperature for 3 days had a moisture content of 8.03%, while dried leaves obtained using fix bed at 40 °C for 4 h had a 6.55% moisture content.

Regarding the three extractions cycles needed to extract the total amount of phenolic compounds in the dried leaves, in the first extraction, 94.87% of the phenolic compounds present were extracted; 5.12% in the second extraction, and in the third extraction there was no detectable phenolic compounds. Based on this, it was decided to carry out a single extraction representing 94.87% of the total phenolic compounds present in the dried leaf. This agrees with other authors [6,7] who also carried out only one extraction.

Regarding the amount of total phenolic compounds present in fresh muicle leaves, fix bed dried leaves (47 °C for 4 h) and leaves dried at room temperature for 3 days (72 h), practically 50% of the total phenolic compounds present in the fresh leaves were lost during any of the drying processes, with no significant difference between them. The remnant phenolic compounds content in the dried leaves were on average 25 mg/g_dw_. Based on this, leaves drying at 47 °C for 4 h were chosen for all experiments.

### 3.2. Best Extraction Conditions for Maximum Phenolic Compounds and Flavonoids Content

Best extraction conditions were estimated using a Box–Behnken design. The optimum variables levels were: temperature 35 °C, ethanol concentration 40%, and extraction time 1 h. Predicted values were PC, 0.333 mg GAE/g_ext_ and FL, 0.200 mg CE/g_ext_ with a desirability value (D) of 0.8124. These predicted conditions were experimentally validated, obtaining 0.334 ± 0.001 mg GAE/g_ext_ for PC and 0.278 ± 0.002 mg CE/g_ext_ for FL. Predicted and experimental results obtained with optimum conditions were very similar.

### 3.3. Bioactive Compounds in Dry Muicle Leaves Using Optimal Extracting Conditions

Table 1 shows the results obtained for the determination of the BC in dried muicle leaves using the Box–Behnken optimum extraction conditions. The PC obtained in this work was lower than that reported by Baqueiro and Guerrero [7], and Sepúlveda-Jiménez et al. [6], but flavonoids and antioxidant capacity content were higher than those reported by Sepúlveda-Jiménez et al. [6], and Baqueiro and Guerrero [7] respectively. These differences may be due to several factors such as the type of soil, temperature, and height over sea level where the plant was grown, the maturity of leaves, and the time of the year they were collected.

There are no reports of the presence of HT and HC compounds in muicle leaves; this is the first report of tannins content in muicle leaves. As can be seen in Table 1, HT content is much higher than CT, about 24 times higher. Anthocyanins were not detectable in muicle leaves, which agrees with the reports of Wrolstad [25] and Baqueiro and Guerrero [7] who did not find the presence of these in their extract.

### 3.4. Determination of Bioactive Compounds Present in Optimized muicle Extract and Concentrated Extract

Table 2 shows the content of all bioactive compounds analyzed present in the extract obtained under optimal conditions, and the concentrated extract (Section 2.5) to obtain in the encapsulated product a concentration of total phenolic compounds similar to those reported by Robert et al. [18], for pomegranate extract and juice.

### 3.5. Muicle Microcapsules Characterization

#### Bioactive Compounds Retention Efficiency

**Figure 1:**
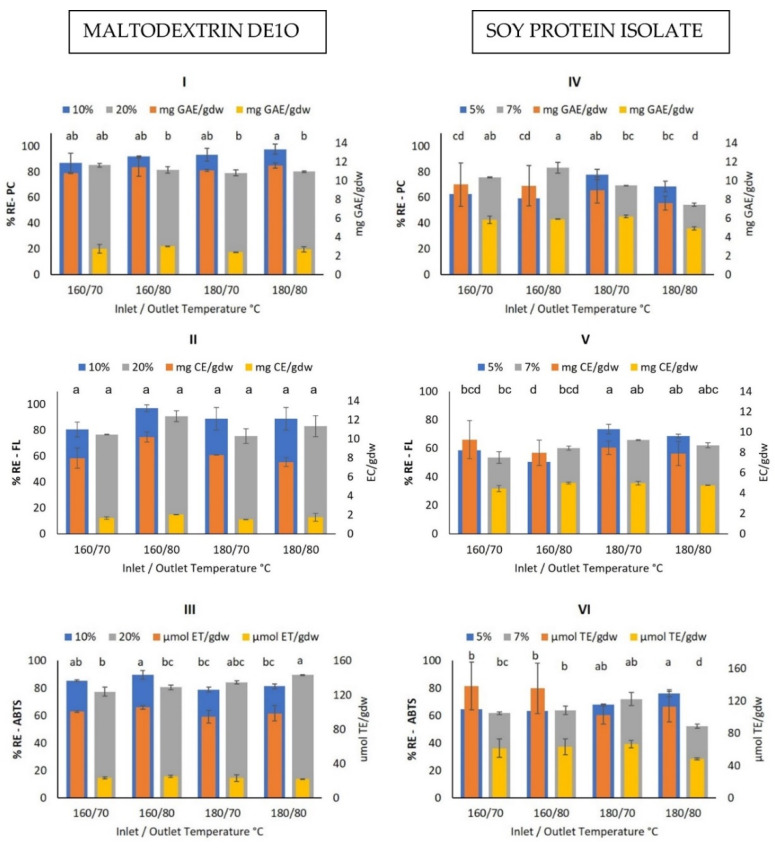
Retention efficiency (%RE) and content of bioactive compounds: PC, FL, and AC (ABTS) in microcapsules with MD DE10 (10% and 20% in drying solution), (**I**), (**II**) and (**III**) respectively; and with SPI as encapsulating agent (5% and 7% in drying solution) (**IV**), (**V**) and (**VI**), respectively. Different letters indicate a significant difference at *p* < 0.05 for retention efficiency.

Retention efficiency (%RE) and content of BC in the microcapsules, were determined for all drying conditions, type, and concentration of encapsulating agent used in this work. An example of the results obtained is presented in Figure 1 where the %RE and content of total phenolic compounds (PC), flavonoids (FL), and antioxidant capacity (AC) determined by ABTS, are shown.

As can be seen in Figure 1 (I), %RE of PC varied from 87.02 ± 7.5% to 97.83 ± 3.84% and 79.35 ± 2.5% to 85.29 ± 1.5% for 10 and 20% MD encapsulating agent concentration in the drying solution respectively, depending on drying conditions (air inlet and outlet temperature). Based on the statistical analysis 10% of MD wall material had a significant effect *(p* < 0.05) on the PC retention, on the other hand, inlet and outlet temperatures, had no significant effect. Regarding PC content in the microcapsules, Figure 1 (I) shows that it ranged from 10.77 ± 0.01 mg GAE/g_dw_ to 11.59 ± 0.28 mg GAE/g_dw_ and 4.89 ± 0.15 to 6.18 ± 0.13 mg GAE/g_dw_ for 10 and 20% MD, respectively, depending on dying conditions.

Regarding the retention efficiency of PC when SPI was used as wall material (Figure 1 (IV)), it varied from 59.26 ± 1.29% to 77.97 ± 3.9% and 54.58 ± 1.2% to 83.33 ± 1.2% for 5% and 7% encapsulating agent concentration respectively, depending on drying conditions. On average the PC retention efficiency with SPI as wall material was lower than that obtained with MD. In the statistical analysis, outlet temperature and wall material concentration had a significant effect (*p* < 0.05) on PC retention obtaining the highest PC retention at 7% wall material and 80 °C outlet temperature. In the same way, the PC content in the microcapsules ranged from 7.59 ± 0.76 to 9.57 ± 2.3 mg GAE/g_dw_ and 4.89 ± 0.15 to 5.88 ± 0.13 mg GAE/g_dw_ for 5% and 7% SPI concentration respectively, depending on drying conditions.

Retention efficiency (%RE) and amount of FL in the microcapsules shown in Figure 1 (II) for MD and Figure 1 (V) for SPI show a similar behavior than PC in the sense that higher %RE of FL were obtained with MD as compared to SPI for all experimental conditions. Statistical analysis for MD as wall material showed that the higher outlet temperature and 10% wall material concentration had a significant effect (*p* < 0.05) on the FL retention. On the other hand, for SPI only the inlet temperature had a significant effect on the retention of flavonoids, obtaining higher FL retention with 180 °C inlet temperature.

Regarding %RE and content of AC determined by ABTS in the microcapsules, Figure 1 (III) for MD and (VI) for SPI, show again that higher retentions of AC (ABTS) were obtained with MD for practically all experimental conditions. The statistical analysis showed that only the outlet temperature was significant for MD (*p* < 0.05) being 80 °C the parameter that had a more negative effect on AC retention. For SPI only 5% wall material concentration was statistically significant (*p* < 0.05).

### 3.6. Best Spray Drying Conditions for Maximum BC Retention

The optimization of the spray drying process to obtain the maximum retention of BC and AC in the microcapsules with both MD and SPI wall materials was made considering the variables mentioned in Section 2.7. The predicted optimum conditions obtained with the software Minitab v. 17, were for MD: 160 °C Ti, 80 °C To and 10% MD concentration, and for SPI: 180 °C Ti, 70 °C To and 5% SPI concentration. These conditions were experimentally validated. Table 3 for MD and 4 for SPI show the predicted and experimental values obtained with the optimum operating conditions.

As can be seen in Table 3 and Table 4, the predicted values are very close to the experimental ones; therefore, the predicted optimum operating conditions can be used with confidence. Although Table 3 shows that MD microcapsules had higher retention efficiencies than SPI microcapsules (Table 4) for all BC analyzed, both microcapsules were tested in the prepared functional food products. This was because maltodextrin, being a water-soluble material, when entering in contact with the product will be rapidly dissolved, releasing the bioactive compounds in the product; whereas SPI, being soluble in alkaline pH, will remain in its original state at the acidic pH of the products, releasing only the BC present on the surface of the microcapsules; therefore, it is expected to mask, at least partially, the bitter taste of the muicle extract.

#### 3.6.1. Particle Size Distribution for the Microcapsules Obtained in Optimal Conditions

**Figure 2:**
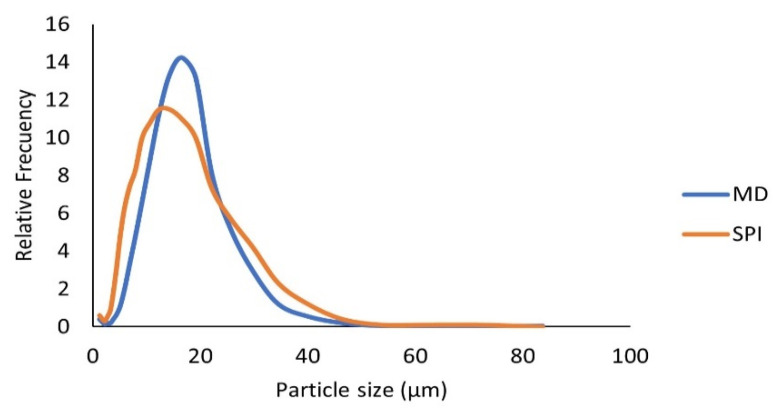
Particle size distribution in muicle extract microcapsules obtained under optimal drying conditions (160–80 °C, 10% wall material concentration for MD and 180–70 °C, 5% wall material for the SPI).

The particle size distribution of microcapsules in the optimum drying conditions is shown in Figure 2. For MD microcapsules, particle size varied between 1.26–50 µm with a Sauter diameter (D [2,3]) of 12.05 ± 0.6 µm and an equivalent sphere diameter (D [3,4]) of 16.2 ± 0.3 µm. For SPI microcapsules, particle size ranged from 1.2–55 μm, having a Sauter diameter (D [2,3]) of 9.2 ± 0.1 µm and an equivalent sphere diameter (D [3,4]) of 14.1 ± 0.8 µm.

As can be seen in Figure 2, SPI microcapsules tend to be a bit smaller than MD microcapsules, which could be due to the smaller amount of wall material used in SPI microcapsules (5% compared to 10% for MD) or the viscosity of the mixture [28]. The results obtained in this work are similar to those reported by Wang and Zhou [29] who obtained particle size of 1–100 μm in spray-dried soy sauce extract powders, using maltodextrin concentrations of 10% to 30%. Moreno, et al. [30] in their work on microencapsulation by spray drying of grape pomace extracts with MD and whey protein isolate, reported values of equivalent sphere diameter (D [3,4]) of 16.2 μm, which is very similar to the ones obtained in this work.

#### 3.6.2. Surface Morphology of Microcapsules Obtained in Optimal Conditions

**Figure 3:**
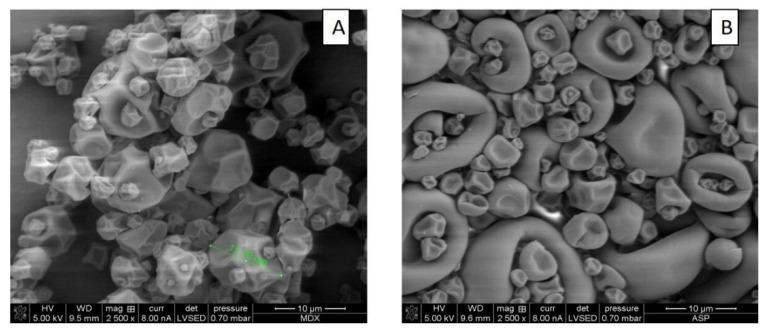
Micrographs obtained by scanning electron microscopy of the muicle extract spray-dried microcapsules with MD (**A**) and SPI (**B**) obtained under optimal conditions.

Micrographs of microcapsules surface morphology obtained through scanning electron microscopy (SEM) at 2500× are shown in Figure 3A for MD and Figure 3B for SPI microcapsules.

Micrographs in Figure 3A for MD microcapsules show wrinkled spherical particles of various sizes; some small particles forming clusters with large particles. No broken particles are seen in the images. Figure 3B for SPI microcapsules show microcapsules of various sizes with a smoother surface, large ones in the form of red blood cells, and smaller ones with a smother wrinkled spherical shape of different sizes [31].

The presence of wrinkled spherical microparticles is attributed to the fact that during drying there is rapid evaporation of water from the particle surface [32]. Greater shrinkage and wrinkling were observed for the more porous wall material. The surface of the dried SPI particles was smoother than the MD particles because they are less porous to water vapor.

### 3.7. Sensory Assessment of Functional Food Products

#### 3.7.1. Yogurt with Added Muicle Extract MD and SPI Microcapsules

**Figure 4:**
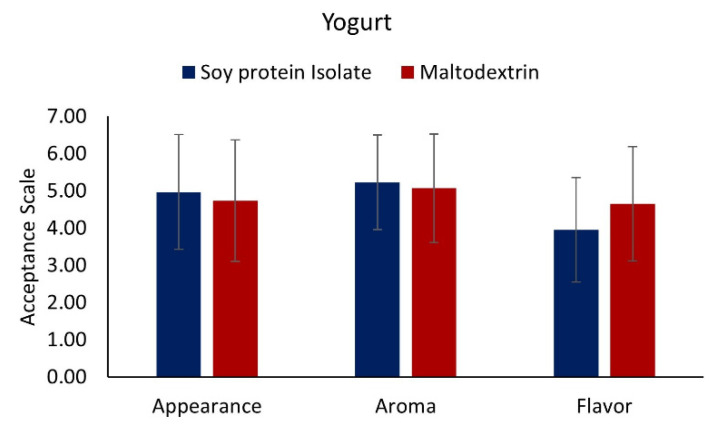
Sensory evaluation of yogurt with added muicle extract MD and SPI microcapsules. 1 = Dislike Very Much, 2 = Dislike Moderately, 3 = Dislike Slightly, 4 = Neither like nor dislike, 5 = Like Slightly, 6 = Like Moderately, 7 = Like Very Much.

**Figure 5:**
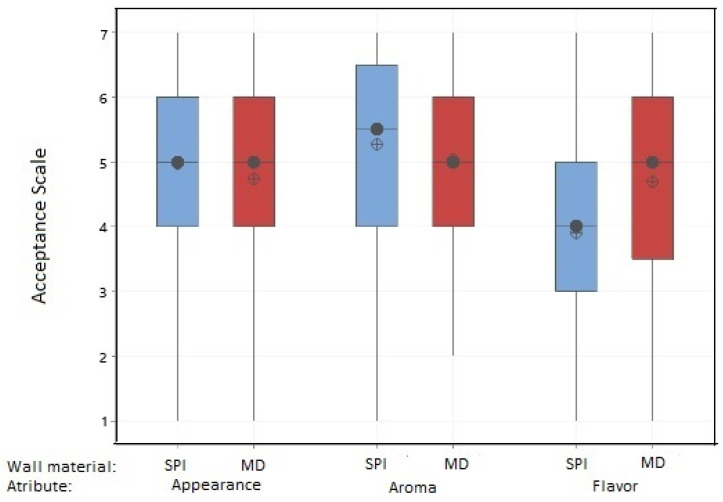
Statistical Analysis (U test Mann-Whitney) for yogurt with added muicle extract MD and SPI microcapsules. 1 = Dislike Very Much, 2 = Dislike Moderately, 3 = Dislike Slightly, 4 = Neither like nor dislike, 5 = Like Slightly, 6 = Like Moderately, 7 = Like Very Much.

Figure 4 shows the results of the sensory evaluation of yogurt added with microcapsules with both wall materials, and Figure 5 the U test Mann-Whitney statistical analysis, for the evaluated attributes (aroma, appearance, and flavor). As shown in Figure 4 for the flavor parameter, yogurt added with both types of microcapsules scored of 4–4.5 (neither like nor dislike-like moderately) being the yogurt with MD microcapsules which obtained a major acceptance from the judges. Figure 5 shows that a significant difference exists (*p* < 0.05) between yogurts with added MD or SPI microcapsules, with yogurt with MD microcapsules being the most preferred by the judges, in which the median for this material obtained a score of 5, having a rating of “like slightly”.

Regarding the aroma attribute for the yogurts with both wall materials, a score of around 5 “like slightly” (Figure 4) was obtained; yogurt with SPI microcapsules showed a slightly better preference by the judges. The U test Mann-Whitney statistical analysis (Figure 5) indicated no significant difference (*p* > 0.05) between yogurts with any of both types of microcapsules, obtaining a score of 5–5.5 “like slightly”.

For the appearance attribute, Figure 4 shows that yogurts with both wall materials obtained almost the same score of about 4.5–4.8 “neither like nor dislike”. Performing the statistical analysis of the results given by the judges, the U test Mann-Whitney statistical analysis (Figure 5), showed that there was no significant difference (*p* > 0.05) between yogurts with both types of microcapsules. The score obtained for the appearance attribute was 5 with “Neither like nor dislike”.

In summary, yogurt added with muicle extract MD microcapsules was better preferred in terms of flavor, obtaining a rating of “like slightly”. In terms of appearance and aroma, there was no significant difference between both materials (*p* > 0.05), obtaining similar ratings of “like slightly”. Although these are acceptable ratings, the formulation of the functional yogurt must be improved in order to obtain higher scores of the evaluated attributes to generate greater acceptance by potential consumers.

#### 3.7.2. Jelly with MD Microcapsules

**Figure 6:**
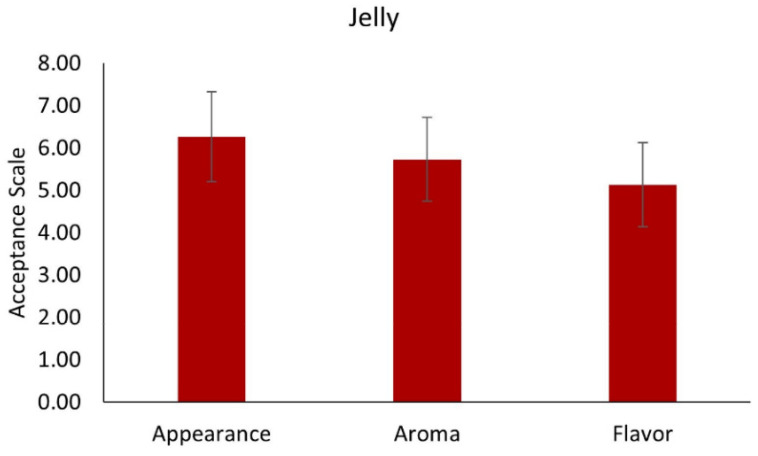
Sensory evaluation of jelly with added muicle extract MD microcapsules. 1 = Dislike Very Much, 2 = Dislike Moderately, 3 = Dislike Slightly, 4 = neither like nor dislike, 5 = Like Slightly, 6 = Like Moderately, 7 = Like Very Much.

Since yogurt with added muicle extract MD microcapsules were the product that had the better scores in preference from the judges, another product (jelly) was prepared by adding MD microcapsules, and a sensory test was also performed. The results of the sensory evaluation for jelly with added MD microcapsules are presented in Figure 6. For the flavor attribute, a score of 5 was obtained (“like slightly”), however, for aroma, the score was close to 6 (between “like slightly” and “like moderately”). Regarding the appearance attribute, the judges scored the jelly with a rating of 6 “like moderately”. Although these are acceptable ratings, the formulation of the jelly must be improved in order to obtain higher scores and greater acceptance by potential consumers.

### 3.8. Preservation of Bioactive Compounds and Antioxidant Capacity during Storage in Manufactured Functional Foods Products

#### 3.8.1. Preservation of Bioactive Compounds in Yogurt Added with Muicle Extract MD and SPI Microcapsules

**Figure 7:**
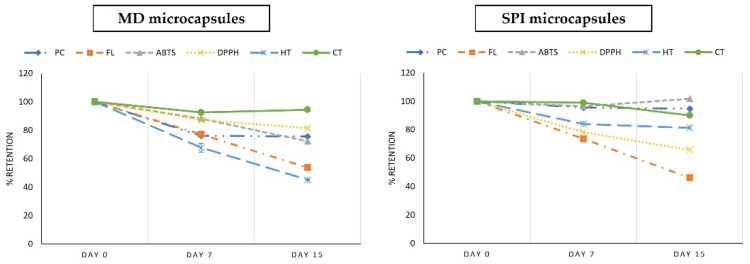
Retention (%) based on day 0 of bioactive compounds (PC, FL, AC (ABTS and DPPH), HT, CT) in yogurt added with muicle extract MD and SPI microcapsules.

Preservation of muicle extract bioactive compounds for 15 days storage, at 4 °C in the dark for yogurt, is shown in Figure 7, expressed as % retention with respect to day 0. As can be seen in this figure, at the end of the storage period (day 15), the bioactive compounds: PC, AC (ABTS), and TH, were better preserved in yogurt with SPI microcapsules. On the contrary, FL and CT showed slightly better preservation in yogurt with MD microcapsules, and AC (DPPH) was considerably better preserved in the latter.

Comparison of means, using the Tukey test, showed that there is a significant difference (*p* < 0.05) between yogurts with MD and SPI, the latter being the one who obtained the better retention of PC with 95% retention, AC (ABTS) with 100% retention and TH with 81% retention. On the contrary, for yogurt with MD microcapsules, the comparison of means, using the Tukey test, showed that FL was better preserved, ending with 54% retention, compared to 46% for yogurt with SPI microcapsules, as well of AC (DPPH) with final retention of 81% compared with 63% for yogurt with SPI microcapsules. For CT, the Tukey test showed no significant difference between the retention with both types of microcapsules.

In summary, for yogurt with added MD or SPI microcapsules, it is difficult to choose which microcapsules was better, since, for MD microcapsules better retention of flavonoids (54%), DPPH (81%) was obtained, however, with SPI microcapsules, retention of phenolic compounds (94%), ABTS (100%) and hydrolyzable tannins (81%) was better. Averaging all BC retention for each type of microcapsules in yogurt, SPI microcapsules retained 75% of BC retention, while MD microcapsules obtained 70% retention of all BC; therefore, based on these values, yogurt with SPI microcapsules is slightly better.

Nevertheless, as shown previously in the sensory evaluation of the prepared functional foods, yogurt with MD microcapsules had better acceptance on the flavor parameter, obtaining a score of “like slightly”; therefore, there are attributes of this product that should be improved to increase preference by the consumer.

#### 3.8.2. Preservation of Bioactive Compounds in Jelly with MD Microcapsules

**Figure 8:**
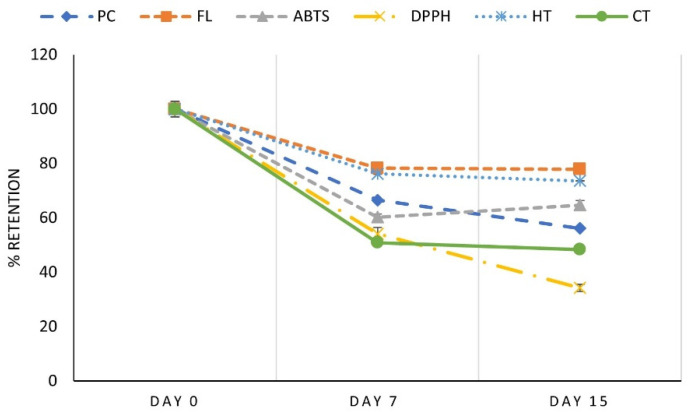
Retention (%) based on day 0 of bioactive compounds (PC, FL, AA (ABTS and DPPH), HT, CT) in jelly added with muicle extract MD microcapsules.

The preservation of muicle extract bioactive compounds for 15 days storage, at 4 °C in the dark for jelly added with MD microcapsules, is shown in Figure 8, expressed as percent retention with respect to day 0. As can be seen in this figure, at the end of the storage period (day 15), the retention of BC was: PC 56%, FL 78%, AC (ABTS) 65%, AC (DPPH) 34%, HT 64%, and CT 48%.

Comparing the retention of BC at the end of the storage for yogurt and jelly added with MD microcapsules, it can be observed that, with the exception of FL (54% in yogurt, 78% in jelly) and HT (45% in yogurt, 64% in jelly), the rest of the BC analyzed were better preserved in yogurt; this could be due to the presence of fat that protects BC from oxidation. Retention of AC (ABTS) in yogurt was 72% compared with 65% in jelly; AC (DPPH) in yogurt was 81% compared to 34% in jelly, and PC in yogurt was 76% compared to 56% in jelly.

In general, the prepared functional foods products with MD as wall material obtained greater acceptance in sensory evaluation, however, when using this wall material in food matrices such as jelly and yogurt, being highly soluble, it did not protect them in an expected way. With SPI as wall material, better retention percentages of bioactive compounds were obtained during storage, obtaining up to 75% average retention. The better protection of phenolic compounds observed in yogurt with SPI microcapsules may be due to the low solubility of soy protein isolate at the acidic pH of yogurt (pH 4), so the capsules maintain their integrity longer, releasing slowly the encapsulated compounds. Based on this, a jelly prepared with SPI microcapsules, hiding the turbidity due to the insoluble microcapsules with the addition of milk, for example, could preserve the BC better than with MD microcapsules in an aqueous matrix. We have done this, and in effect, better BC retention was obtained but as happened with yogurt, the acceptance in flavor was not very good, so an improvement in the formulation has to be done.

## 4. Conclusions

With the optimal extraction conditions obtained with a Box–Behnken experimental design, it was possible to extract 95% of bioactive compounds present in muicle leaves. Muicle leaves were shown to have a high amount of hydrolyzable and condensable tannins that have not been reported so far.

Using maltodextrin DE10 and soy protein isolate as wall material, it was possible to obtain muicle leaves extract microcapsules, with very good retention of bioactive compounds. These microcapsules when used in the preparation of functional food gave good preservation of the bioactive compounds during 15-day storage, but the sensory evaluation for flavor, appearance, and aroma was only fair, “like slightly”. In general, products with muicle extract MD microcapsules had a better acceptance (*p* < 0.05) in the flavor parameter than the products with SPI microcapsules, but retention of BC during storage was better for products with SPI microcapsules.

Results of this work show that muicle extract microencapsulated by MD or SPI as wall material can be considered as a promising source of bioactive compounds for the preparation of functional foods, but improvement in the formulation is required to increase their sensory evaluation scores and acceptance by the consumer.

## Figures and Tables

**Table 1:** Bioactive compounds in dried muicle leaves using optimal extraction conditions.

Determination	Content
Phenolic compounds (PC)	27.05 ± 0.020
Flavonoids (FL)	13.88 ± 0.20
Antioxidant Capacity ABTS (AC)	2.7 ± 0.35
Antioxidant Capacity DPPH (AC)	0.55 ± 0.39
Hydrolyzable tannins (HT)	322.31 ± 1.99
Condensed tannins (CT)	12.85 ± 0.33
Monomeric anthocyanins (MA)	Not detectable

Results are expressed as the mean ± standard deviation. PC: mg GAE/g_dw_, FL: mg CE/g_dw_, HT: mg TAE/g_dw_, CT: mg CE/g_dw_, AC: µmol TE/g_dw_.

**Table 2:** Bioactive compounds in optimized muicle extract and concentrated extract.

Determination	Optimized Muicle Extract	Concentrated Muicle Extract
Phenolic compounds (PC)	0.33 ± 0.00	2.00 ± 0.03
Flavonoids (FL)	0.27 ± 0.00	1.76 ± 0.00
Antioxidant Capacity ABTS(AC)	4.10 ± 0.14	18.63 ± 0.16
Antioxidant Capacity DPPH (AC)	1.19 ± 0.09	2.11 ± 0.09
Hydrolyzable tannins (HT)	8.92 ± 0.04	41.67 ± 0.38
Condensed tannins (HC)	0.23 ± 0.04	1.24 ± 0.01

Results are expressed as the mean ± standard deviation. PC: mg GAE/g_dw_, FL: mg CE/g_dw_, HT: mg TAE/g_dw_, TC: mg CE/g_dw_, AC: µmol TE/g_dw._

**Table 3:** Experimental and predicted values for all studied variables, for MD microencapsulated muicle leaves extract obtained with the optimum operating conditions (2^3^ factorial design).

Response Variables BC	Predicted Values Experimental Values % Retention Efficiency	
PC	92.02	86.82 ± 0.87
FL	97.07	90.76 ± 5.72
AA (ABTS)	89.83	85.22 ± 3.63
AA (DPPH)	82.67	82.18 ± 3.62
HT	90.23	91.31 ± 1.99
CT	92.28	95.75 ± 3.37

Experimental values are expressed as the mean ± standard deviation of the analysis in duplicate drying runs. PC: phenolics compounds, FL: Flavonoids, HT: hydrolyzable tannins, CT, Condensed tannins, AC: antioxidant capacity.

**Table 4:** Experimental and predicted values for all studied variables, for SPI microencapsulated muicle leaves extract obtained with the optimum operating conditions (2^3^ factorial design).

Response Variables BC % Retention Efficiency	Predicted Values Experimental Values % Retention Efficiency	
PC	77.48	82.95 ± 1.57
FL	73.38	74.08 ± 1.99
AA (ABTS)	68.42	70.11 ± 2.10
AA (DPPH)	69.95	70.98 ± 0.91
HT	72.44	72.88 ± 1.49
CT	60	65 ± 1.18

Experimental values are expressed as the mean ± standard deviation of the analysis in duplicate drying runs. PC: phenolics compounds, FL: Flavonoids, HT: hydrolyzable tannins, CT, Condensed tannins, AC: antioxidant capacity.
